# Metagenomic discovery of lipases with predicted structural similarity to *Candida antarctica* lipase B

**DOI:** 10.1371/journal.pone.0295397

**Published:** 2023-12-06

**Authors:** Nongluck Jaito, Nattha Kaewsawat, Suthathip Phetlum, Tanaporn Uengwetwanit

**Affiliations:** National Center for Genetic Engineering and Biotechnology (BIOTEC), National Science and Technology Development Agency (NSTDA), Pathum Thani, Thailand; Weizmann Institute of Science, ISRAEL

## Abstract

Here we employed sequence-based and structure-based screening for prospecting lipases that have structural homolog to *Candida antarctica* lipase B (CalB). CalB, a widely used biocatalyst, was used as structural template reference because of its enzymatic properties. Structural homolog could aid in the discovery of novel wild-type enzymes with desirable features and serve as a scaffold for further biocatalyst design. The available metagenomic data isolated from various environments was leveraged as a source for bioprospecting. We identified two bacteria lipases that showed high structural similarity to CalB with <40% sequence identity. Partial purification was conducted. In comparison to CalB, the enzymatic characteristics of two potential lipases were examined. A candidate exhibited optimal pH of 8 and temperature of 50°C similar to CalB. The second lipase candidate demonstrated an optimal pH of 8 and a higher optimal temperature of 55°C. Notably, this candidate sustained considerable activity at extreme conditions, maintaining high activity at 70°C or pH 9, contrasting with the diminished activity of CalB under similar conditions. Further comprehensive experimentation is warranted to uncover and exploit these novel enzymatic properties for practical biotechnological purposes.

## Introduction

Enzymes are natural catalysts having the ability to accelerate chemical reactions with precision and efficiency. Their properties have led to significant advancements in industrial processes, promoting both economic and ecological sustainability. As industries evolve and demand for sustainable and efficient processes increases, there is a constant need for new and improved enzymes that can address specific challenges and optimize existing processes.

Lipases are an important biotechnological biocatalyst due to their capability to facilitate numerous reactions such as esterification, transesterification and acidolysis [1, 2]. The multifunctional properties of lipase enzymes can be attributed to their diverse and complex structures [3]. These reactions have numerous applications in industries such as laundry, food, chemical, and pharmaceutical industries [4–6]. The global lipase market is expecting to grow from 2022 at a compound annual growth rate of 5.6% by 2032 [7]. One of the most used lipase is the lipase B (CalB) from yeast *Candida antarctica* (currently named *Moesziomyces antarcticus*) [1]. CalB shows broad substrate specificity, high enantioselectivity, tolerance to organic solvents and thermal stability [8–10]. Hence, CalB is a reliable biocatalyst for industrial scale.

Sequence-based enzyme discovery by mining metagenome and genome has been proved in the finding of novel enzymes. Many studies have investigated novel Cal-B lipases from fungal genome by using sequence homology and conserved domains or motifs to CalB. Genome-based bioprospection identified *Ustilago maydis* lipase 2 (Uml2) [11], *Pseudozyma antarctica* lipase (PlicB) [12] and *Ustilago hordei* lipase (UhL) which showed 66%, 30% and 76% sequence identity to CalB, respectively. Identifying putative enzymes on genome based on sequence homolog yields a handful of target enzymes for further characterization. However, finding genes in metagenomic data results in a higher number of target enzymes depending on similarity cutoff. It can be difficult to choose a promising enzyme in the immensity of generated data. The lesser similarity percentage between the known enzymes and newly sequences have been used to discovery of numerous novel enzymes [13–15]. Even if a certain enzymatic function could be inferred based on sequence homology or conserved sequences, no information is obtained regarding its applicability in terms of stability, efficiency, or specificity. To improve the selection process of applicable enzymes, structure-based screening could offer a potentially more reliable approach to infer protein function as structures are three to ten times more conserved that sequences [16]. Moreover, proteins with similar structural conformations often exhibit comparable physical properties, such as solvent tolerance, temperature stability, and pH adaptability [17, 18]. Thus, finding candidate enzymes having structural similarity with the targeted enzymes would provide initial suitable biocatalysts for industrial development.

In this study, we report in silico mining of published metagenomic data to discover bacteria lipases with similar structural CalB. We leveraged the availability of metagenomic data isolated from various environments. Metagenomics are largely focuses on the diversity, structure of microorganisms and targeted bioprospecting [19, 20]. Thus, these published metagenomic data remains largely unexplored and presents an excellent source for bioprospecting. The present study reported the identification and functional characterization of two bacterial lipases that predictively have structural resemblances to fungal lipase CalB.

## Materials and methods

### Metagenomic analysis

Metagenomic sequences from diverse environments used in this work were obtained from the publicly available database (accessions: PRJEB12327, PRJEB18567, PRJEB14874, PRJEB14893, PRJDB7293, PRJEB10054, PRJNA335670, PRJNA339844, PRJNA419239, PRJNA431961, PRJEB14823, PRJEB15175, PRJEB14718, PRJEB14821, PRJEB20578, PRJEB22193, PRJEB9208, PRJNA209710, PRJNA244670, PRJNA277916, PRJNA299404, PRJNA76185, PRJEB14880, PRJEB14900, PRJEB5246, PRJNA371432, PRJNA391943). The sequencing reads were processed to remove low quality bases (Phred quality score < 20) and adaptor sequences using Trim Galore [21]. Clean reads from each study were subjected to metagenomic analysis in OmicsBox software [22]. Metagenomic assembly of the reads was performed by MetaSPAdes [23]. Prodigal [24] was used to predict protein-coding gene. Functional annotation of predicted proteins was carried out using BLAST [25] against customized lipase database, EggNOG-Mapper [26] and PfamScan [27]. The customized lipase database is a collection of lipase protein sequences retrieved from the lipase engineering database [28], protein data bank (PDB) [29] and GenBank [30].

### Structural similarity search

Bacterial lipase-like sequences were aligned to lipase B from *Candida antarctica* (PDB: 1TCA, 5A6V) [31]. Three-dimensional structure of high sequence similarity was predicted using AlphaFold2 [32] in google colab. Visualization and analysis of protein structure superposition was applied to identify similarities of protein folds using MOE [33].

### Phylogenetic analysis

The protein sequences for families of bacterial lipolytic enzymes previously classified by Kovacic et al. [34] were obtained from Uniprot [35]. An amino acid alignment of lipases and metagenomic candidate sequences was generated using ClustalW [36]. This alignment was then imported into the phylogenetic analysis program MEGA [37] using the Maximum Likelihood method and JTT matrix-based model [38].

### Molecular dynamics (MD) simulation studies

MD simulation was carried out using Amber22 [39]. AMBER ff19SB force field was assigned for protein. The structure was solvated in TIP3P water box extending 10 Å and neutralized with Na+/Cl-. The structures were energy-minimized in 2 stages. In the first stage, minimization of water molecules was carried out using 1000 cycles of steepest descent and 3000 cycle of conjugate gradient. The second step, minimization of entire system used 1000 cycles of steepest descent and 2000 cycle of conjugate gradient and then heated gradually from 0 to 323 K in 100 ps. All restraints were removed for the production stage. The simulation time step was 2 fs/iteration with SHAKE algorithm [40] at 323 K for 200 ns.

### Gene cloning and construction of expression plasmid

Commercial CalB was purchased from Siam Victory (Thailand) for biochemical characterization compared to CalB produced in *Escherichia coli* BL21DE3. Plasmid pET22b and pET28a were purchased from GenScript (New Jersey, U.S) and used as the expression vector. A gene encoding CalB from *Candida antarctica* and candidate lipases (SeqA and SeqB) were subjected to codon optimization to enhance protein expression in *E*. *coli* BL21DE3. All genes were synthesized by GenScript without the N-terminal signal peptide. The CalB gene fused with the pelB signal sequence at its N-terminus between the *Nde*I and the *Hind*III sites of pET22b, while the metagenomic lipases were cloned into pET28a at the same restriction site. The constructed plasmids were transformed into *E*. *coli* BL21DE3 for lipase expression.

### Expression and purification of recombinant proteins

The *E*. *coli* BL21DE3 host cell harboring lipase gene were initially inoculated into 5 mL of Luria-Bertani medium (LB) supplemented with 100 μg/mL ampicillin or 50 μg/mL kanamycin for pET22b or pET28a, respectively. The cells were incubated at 37°C, 200 rpm for 16 h, then re-inoculation of 3 mL overnight grown culture into a fresh 3,000 mL LB and incubation at 37°C, 200 rpm until OD600 reached 0.4–0.6. The IPTG was added to the culture in a final concentration of 0.2 mM and expressed at 16°C, 200 rpm for 20 h. After induction, the cells were harvested by centrifugation at 4°C, 8,500 rpm for 5 min, resuspended with 5 mL of ice-cold 50 mM Tris-HCl buffer (pH 8.0), and disrupted by sonication. Afterward, the cell lysates were centrifuged at 12,000 rpm at 4°C for 15 min. Partial purification of enzymes was performed [41]. Briefly, the crude enzyme extract (supernatant) was filtered through a 0.2 μm filter and applied to a HisTrap High-Performance column (HisTrap HP column, GE-Healthcare) which equilibrated with 10 column volumes (CVs) of equilibration buffer (20 mM Tris-HCl buffer, pH 8.0 containing 0.1 M NaCl and 20 mM imidazole). The unbound proteins (F1 and F2) were washed with 5 CVs of equilibration buffer and the bounded proteins were eluted using a stepwise imidazole elution from 50 to 500 mM in the equilibration buffer. After that, the eluted protein fractions were identified for target protein by using 12% SDS-PAGE. The target protein fractions were pooled and dialyzed against 50 mM Tris-HCl buffer (pH 8.0) for overnight at 4°C. All steps of purification were performed at 4°C. The next steps involved further processing the partially purified lipase CalB eluted with 100 mM imidazole, metagenomic lipase SeqA eluted with 100 mM imidazole, and metagenomic lipase SeqB eluted with 250 mM imidazole. Total concentration of eluted proteins was determined and biochemical characterization including optimum temperature, optimum pH, and substrate specificity was conducted.

### Determination of enzyme activity and substrate specificity

Lipase activity was determined by a colorimetric method using the p-nitrophenyl hexanoate (pNP-C6) as substrate with some modifications from the previous report [42]. The reaction mixture was carried out in 1 mL consisting of 50 mM Tris-HCl buffer (pH 8), 0.4% v/v Triton X-100, 1 mM pNP-C6 dissolved in acetonitrile, and 50 μL of appropriate enzyme concentration. After incubation at 50°C for 10 min, and the reaction was stopped by adding 300 μL of 0.1 M Na2CO_3_ (final concentration of 23 mM). The reaction mixture was centrifuged at 12,000 rpm for 1 min, and 100 μL of the reaction was validated in a 96-well plate for the absorbance at 405 nm. A standard curve of the p-nitrophenol (pNP) chromophore was established to determine the lipase activity. Three independent assays were conducted. One unit of lipase activity is defined as the amount of enzyme that can liberate 1 μmol of pNP from substrate per min.

Chain-length specificity was measured with pNP-esters containing variable acyl chain lengths using p-nitrophenyl acetate (pNP-C2), p-nitrophenyl butyrate (pNP-C4), p-nitrophenyl hexanoate (pNP-C6), p-nitrophenyl octanoate (pNP-C8), p-nitrophenyl decanoate (pNP-C10), p-nitrophenyl dodecanoate (pNP-C12), and p-nitrophenyl palmitate (pNP-C16) purchased from Sigma (St. Louis, MO, USA). The substrate and enzyme were mixed and assayed at 50°C, pH 8 for 10 min. An experiment control was prepared using the same procedure, excluding the addition of nay enzyme to the reaction mixture.

### Effect of pH and temperature on lipase activity

To assess the influence of temperature and pH on the hydrolytic activity of the lipase, the substrate was mixed with the 50 μL of enzyme (1.01 ± 0.08 mg/mL of CalB; 0.18 ± 0.02 mg/mL of SeqA; 0.14 ± 0.007 mg/mL of SeqB). Effect of temperature on lipase activity was determined at 30, 40, 50, 55, 60, and 70°C with pNP-C6 as substrate in Tris-HCL (pH 8.0). The impact of pH on enzyme activity was studied at a constant temperature of 50°C, employing various buffer systems with a concentration of 50 mM. These buffers encompassed sodium citrate buffer spanning pH 4.0–5.0, potassium phosphate buffer across pH 6.0–7.0, and Tris-HCl buffer ranging from pH 8.0–9.0. A parallel experiment control was set up following the same procedure, excluding the addition of any enzyme to the reaction mixture. All experiments were repeated three times. The effect of temperature and pH on lipase activity was expressed by relative enzyme activity. The maximum enzyme activity was determined to be 100%.

## Results and discussion

### Discovery of Cal-B like lipases

Twenty-seven metagenomic datasets from diverse environments were selected to maximize genetic variability. Homologous sequence searching was first employed to identify candidate lipases. More than 8 million assembled metagenomic contigs were first screened against the custom lipase database. The sequence similarity screening gave 10,939 bacterial lipases. To focus our efforts on identifying potentially untapped and unexplored lipases, the bacterial metagenomic sequences showing a significant match (>90% similarity) with lipase sequences obtained from the PDB were excluded. This decision was made to avoid the possibility that these lipases have already been extensively studied or utilized in previous research.

In our study, we attempted to screen for lipases resembling CalB through structural analysis. The crystal structure of CalB was used as reference structure because of its excellent enantioselectivity, versatility in substrates, thermal stability, and stability in organic solvents [43]. Utilization of its structural features could help to discover effective biocatalysts for commercial exploitation. As structure prediction required significant computational resources, similarity to CalB was used to filter a handful set of metagenomic lipases for structure prediction. Typically, similar sequences tend to fold into similar structures. Nevertheless, our interest was in the proteins that exhibit dissimilar sequences but still fold into similar structures. Metagenomic sequences annotated as lipases were re-aligned against CalB. Lipases showed less than 40% sequence identity to CalB were subjected for structure prediction. This threshold was set up based on our observations in crystal structures of lipases. There is a notable diversity among lipase structures, exhibiting comparatively low sequence similarity across lipases from different organisms (S1 Fig). Despite the general diversity in lipase structures, some lipases exhibit partial structural similarities with CalB (S2 Fig). In our study, some metagenomic sequences showed sequence identity with CalB were less than 30% which were also known as twilight-zone proteins [44] (S1 Table and S3 Fig). Structure prediction of twilight-zone proteins using homology modeling is not a reliable accurate model [45]. Hence, AlphaFold, a template-free prediction of protein structure, was employed due to its capability to provide more reliable predictions in such challenging scenarios [46]. The predicted structures were visualized and manually curated based on their overall structural similarity.

**Fig 1:**
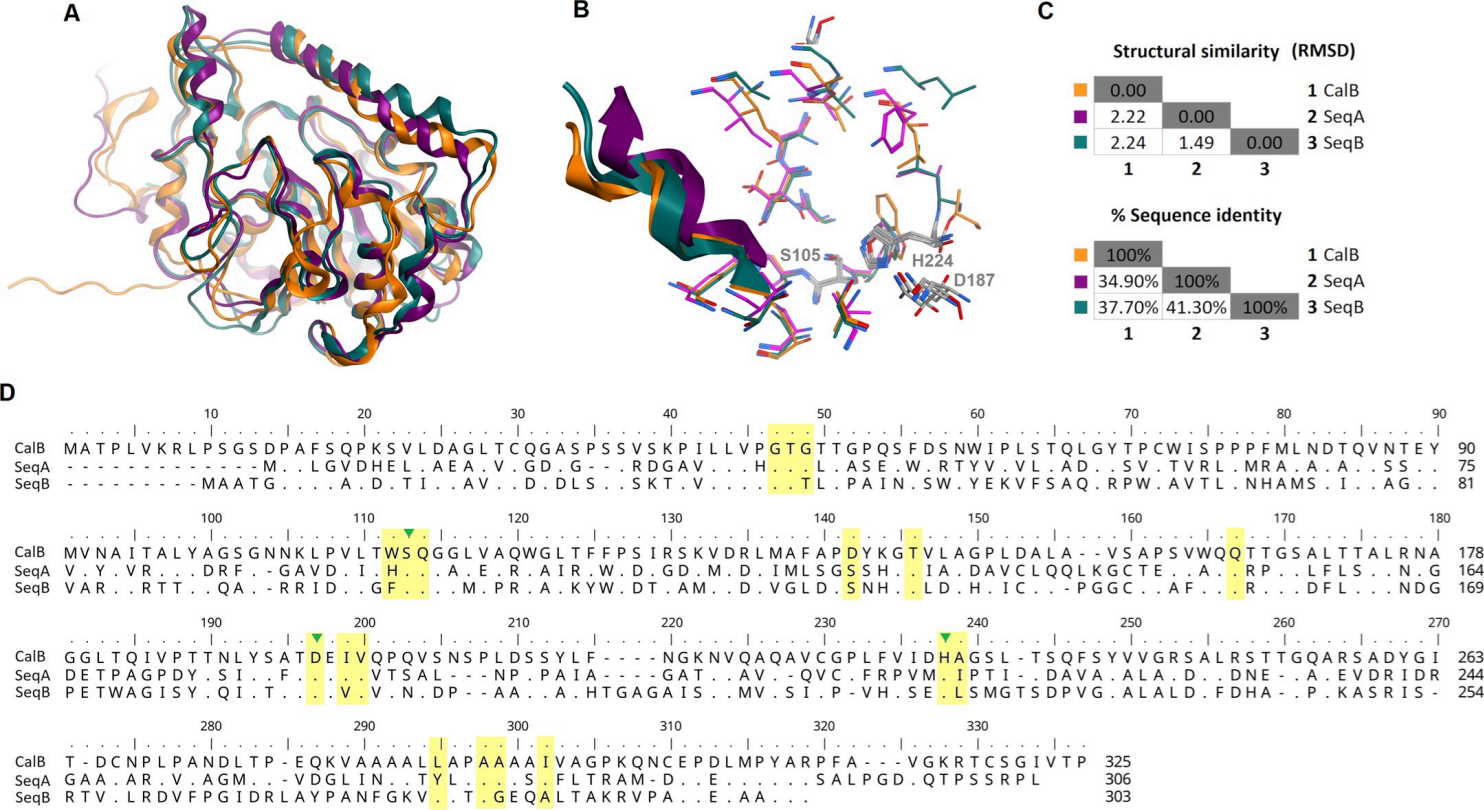
Overall structures and sequences of CalB and candidate lipases. (A) Superposition of CalB (orange) and the lipase structural models of SeqA (purple) and SeqB (teal). The models of SeqA and SeqB were generated by AlphaFold2. The image was created by MOE software. (B) binding site of lipases (C) Similarity matrix (D) Sequence comparison of CalB, SeqA and SeqB. Dots represent identical amino acids. Dashes indicate gaps introduced to maximize the alignment. Amino acid residues located at binding sites are highlighted in yellow box. Green triangles denote catalytic triad. The software BioEdit [48] was used to create the image of sequence alignment.

Among the curated lipases, we selected two candidates named SeqA and SeqB. Upon superimposition of the candidate lipases and CalB, the overall structures and binding site were similar (Fig 1A and 1B) with the root mean square deviation (RMSD) of less than 2.5 Å (Fig 1C). The results suggested the candidate lipases share high structural similarity with CalB even though SeqA and SeqB showed 34.90% and 37.70% of protein identity with CalB, respectively (Fig 1C). The selected lipases also showed same conserved catalytic triad consisting of Ser, His and Asp within the binding pocket [47] (Fig 1B and 1D). Nevertheless, preference of substrate binding might be different as there are variant amino acids in the binding site (Fig 1B). The candidate sequences were aligned with whole genome shortgun contigs using online BLAST (https://blast.ncbi.nlm.nih.gov/). The candidate SeqA and SeqB were 100% identity at amino acid level with *Nocardioides acrostichi* strain CBS4Y-1 Scaffold2 and *Salinisphaera* sp. metagenomic assembled genome, respectively. It confirmed that these lipases belong to the bacteria.

**Fig 2:**
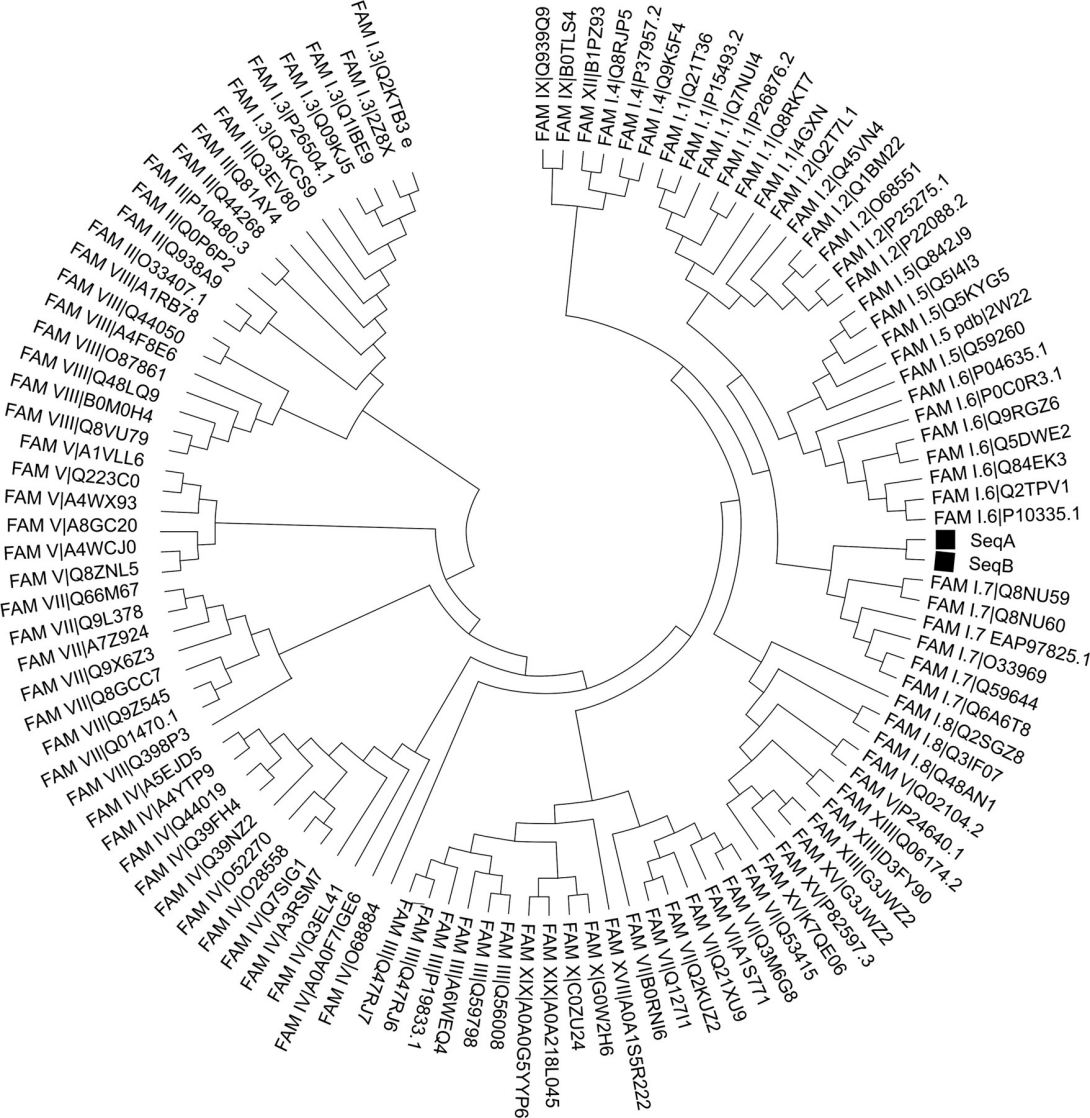
Phylogenetic analysis. Maximum likelihood tree of candidate lipases (SeqA and SeqB) and representative bacterial family lipase proteins.

A phylogenetic tree analysis was conducted to analyze the diversity among the metagenomic lipases and representative known bacterial lipolytic enzymes [34]. The results showed that metagenomic lipases were closely related to subfamily 1.7 (Fig 2). All the referent lipases in family 1, to which subfamily 1.7 belongs, have been characterized as true lipases. True lipases are lipases can hydrolyze water insoluble substrates [49]. The close clustering of the metagenomic lipases suggests that they share common ancestry and may possess similar functional properties to the well-characterized lipases in this family. Hence, it could be implied that these metagenomic lipases are true lipases.

**Fig 3:**
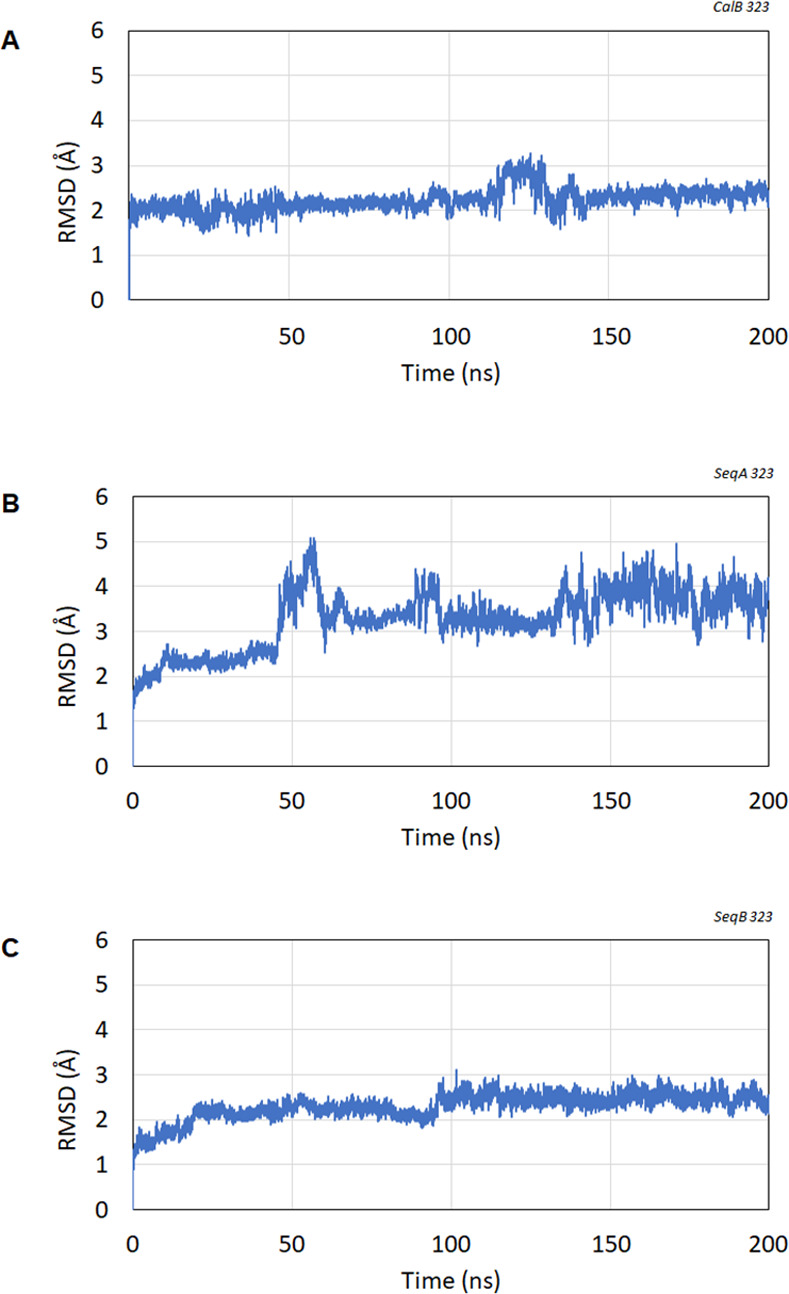
Root means square deviation (RMSD) for 200 ns MD simulation runs at 50°C (323 K). MD production run for each protein (A) CalB (B) SeqA (C) SeqB.

While the predictive structures showed high accuracy that most regions exhibited a per-residue confidence metric (pLDDT) >90, the MD simulation was also conducted to evaluate structural stability at optimum temperature of CalB (50°C) [50]. The RMSD of CalB and SeqB remained stable at below 2.5 Å during the whole simulation period (Fig 3). The results indicate the conformational stability of the structures. The MD simulation of SeqA was stable for the first 40 ns simulations time with RMSD < 2.5 Å, after that the protein SeqA has undergo large conformational change. The results suggest SeqA might be less stable at this temperature in comparison to CalB or SeqB. To further characterize lipase and validation of their properties, gene cloning and biochemical assay were conducted.

### Expression and enzymatic properties of metagenomic lipases

The genes encoding lipases proteins were cloned into expression vector and confirmed successful construction. Partial purification of enzymes was performed. SDS-PAGE analysis showed partially purified protein target protein as a target protein band of ∼35 kDa with significantly higher intensities than the other bands (S4–S6 Figs). Expression of recombinant lipases in heterologous host commonly resulted in the formation of inclusion bodies [51, 52]. The incompatibility could be arisen from several factors such as promoter recognition, inefficient protein translation, and the absence of essential post-translational modifications needed for protein activation [53–56]. Improvement of recombinant lipase production will be further explored.

**Fig 4:**
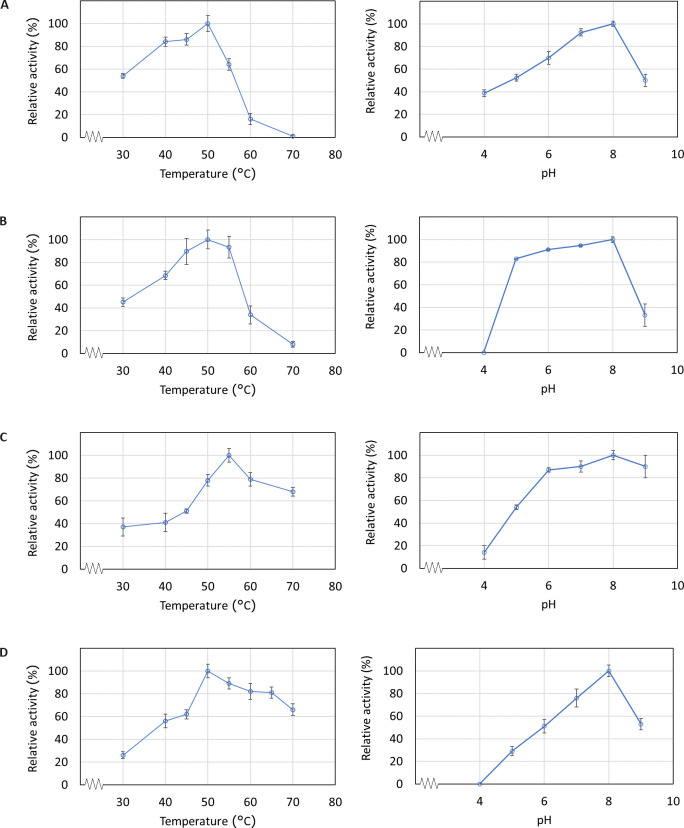
Effect of temperature and pH on enzyme activity. Relative activity of (A) CalB (B) Commercial lipozyme CalB from Siam Victory Chemicals (C) SeqA and (D) SeqB. Enzyme activity was measured at each temperature/pH under standard assay conditions. Data are averages from triplicate experiments.

The activity of CalB (recombinant CalB and commercial CalB) and candidate enzymes were tested at a temperature range of 30–70°C at pH 8.0. The results showed that CalB remained active (≥ 60% relative activity) at a temperature range of 35–55°C. Candidate SeqB showed highest activity at 50°C as similar as CalB whereas the highest lipases performance of SeqA was at 55°C (Fig 4). At 70°C, relative activity of candidate lipase SeqA and SeqB remained over 60% whereas remaining activity of CalB was nearly inactive. The results suggest that candidate lipases could be good candidates for thermophilic conditions. It is noteworthy that lipase activity depends on the substrate and activity determination condition [57]. Hence, optimum temperature reported here were slightly different than previous studies. For example, at pH 7.4, an optimum temperature of 55°C of for CalB was reported [58] while at pH 8.0, purified CalB demonstrated an optimum temperature of 52°C [42].

The activity of enzymes is influenced by pH because the pH of the environment affects the ionization state of amino acid residues. The effect of pH on lipase activity was determined by measuring the activity at 50°C over the pH range of 4.0–9.0. CalB was observed to be active (>60% relative activity) in the pH 6–8 and an optimum pH for lipase activity was observed to be 8 (Fig 4). Similarly, both partially purified lipases, SeqA and SeqB, displayed an optimum pH of 8 mirroring the behavior of CalB (Fig 4). However, their activity spanned in slightly different pH ranges. SeqA exhibited notable activity between pH 6 and 9 while SeqB demonstrated activity within the pH range of 7 to 8. Interestingly, SeqA showed remarkable relative activity surpassing 80% at pH 9, in contrast to the lower than 60% activity observed for CalB and SeqB at this pH.

Given its exceptional performance at high temperatures and particularly at an alkaline pH of 9, SeqA could potentially serve as a superior enzyme in applications requiring elevated temperatures or alkaline environments. This unique characteristic positions SeqA as a compelling candidate for various industrial or biotechnological processes where CalB might be less effective or unsuitable.

**Fig 5:**
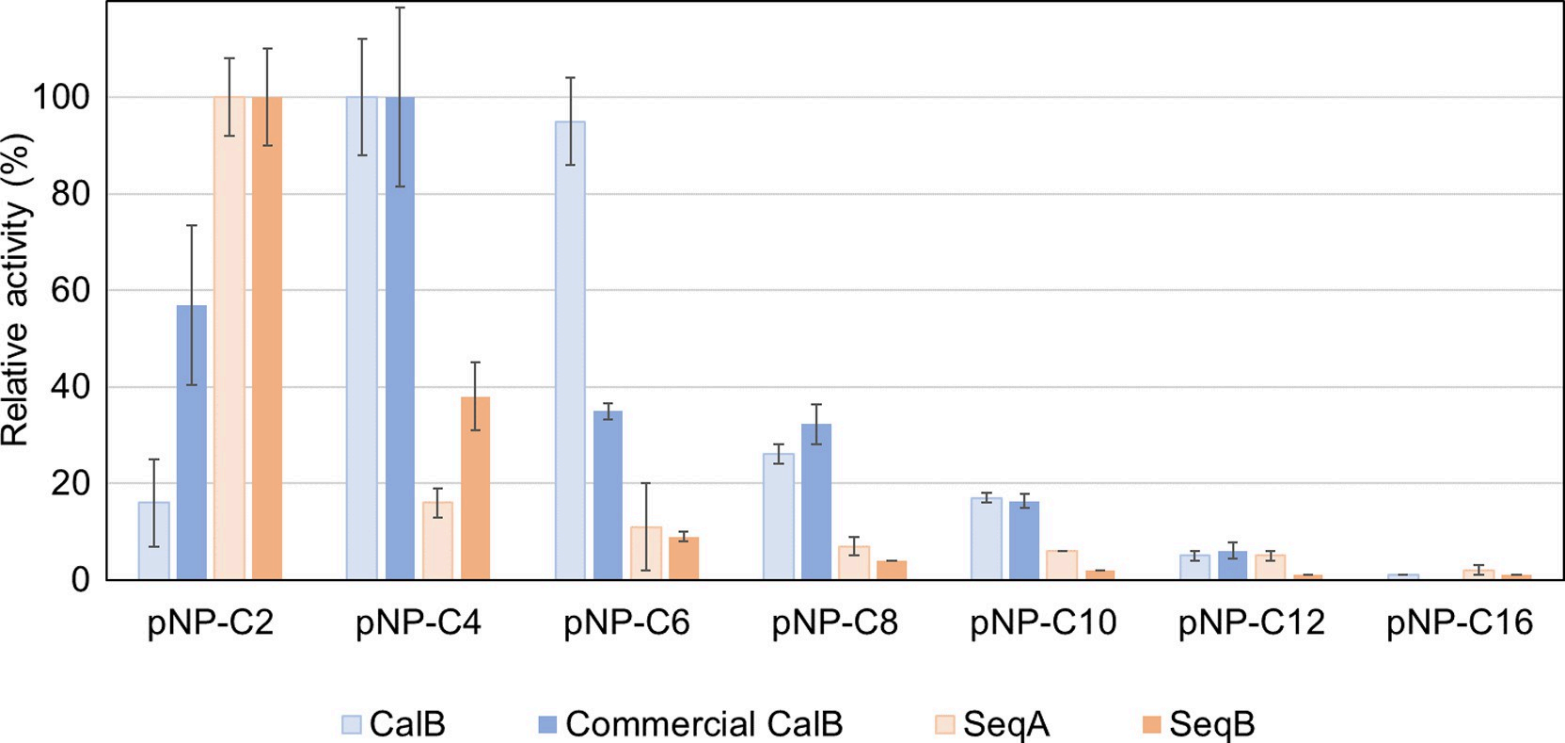
Substrate specificity of lipase assayed using p-nitrophenyl esters (pNPs) of different carbon chain length. Substrate specificity of CalB, Commercial lipozyme CalB from Siam Victory Chemicals, SeqA and SeqB. Data are averages from triplicate experiments.

To identify the specificity of candidates, the enzymatic activity was evaluated as a function of different chain length of pNP substrates. The results showed CalB and candidate lipases preferentially hydrolyzed short acyl chains (Fig 5). Candidate SeqA and SeqB showed highest activities against pNP acetate (pNP-C2) whereas the highest relative activity of CalB was observed against pNP butyrate (pNP-C4). Activity reduced as the acyl chain length increased. The differences in substrate preferences might be due to specific amino acid substitutions within the binding pocket (Fig 1B).

Sequencing data has become more affordable and accessible thus the use of sequence-based screening to mine metagenomic data for novel enzymes has increased. Sequence-based screening has the drawback that it may not be effective when the novel enzyme only has low similarities to known enzymes or when the sequence similarity does not match to a function. Because structure conserves information more than sequences do, structure-based screening could aid in the discovery of novel wild-type enzymes with desirable features and serve as a scaffold for further biocatalyst design. In this study, we integrated sequence-based and structure-based screening to discover structural CalB-like lipases which were derived from bacteria. The candidate lipases have some similar enzymatic properties to CalB, but both exhibit intriguing characteristics. Further study on the relationship between structure and enzymatic properties should be useful for biocatalysis engineering. While AlphaFold is an acceptable and highly accurate protein structure prediction method, experimental 3D protein structure determination should be considered for validation. Optimization, production, and purification processes should be further conducted to fully ascertain their biochemical properties and assess their feasibility for various biotechnological applications.

## Supporting information

S1 Table Example of metagenomics proteins having sequence identities with CalB lower than 30%. (DOCX)

S1 Fig Sequence similarity of lipases compiled from the protein data bank (PDB). Data on the upper right presents the sequence identity and data on the bottom left presents the sequence similarity. The multiple sequence alignment was created by MatGAT2. The sequence no.1 to 15 are lipases from eukaryotes and no.16 to 31 are lipases from prokaryotes.
(TIF)

S2 Fig Structural superposition of CalB (PDB: 5A6V open form) and other lipases sharing structurally similar regions. Lipases from *Neosartorya fumigata* (PDB: 6IDY) and *Lasiodiplodia theobromae* (PDB: 7V6D) are presented in grey. CalB structure is presented in orange.
(TIF)

S3 Fig Structural superposition of CalB and metagenomic lipases that share sequence identity less than 30%. CalB structure is presented in orange and predicted structures of metagenomic lipases are presented in cyan.
(TIF)

S4 Fig SDS-PAGE analysis of lipase from *Candida antarctica* (CalB). Lane M: protein molecular weight marker; Lane F1 and F2: unbounded protein fractions; Lane E1: Eluted protein with 100 mM Imidazole. The partially purified enzyme was indicated by arrow. The total protein concentration of eluted CalB protein was 5.06 ± 0.41 mg.
(TIF)

S5 Fig SDS-PAGE analysis of lipase from metagenomic SeqA. Lane M: protein molecular weight marker; Lane C: crude enzyme extract; Lane F1 and F2: unbounded protein fractions; Lane E1: Eluted protein with 100 mM Imidazole. The partially purified enzyme was indicated by arrow. The total protein concentration of eluted protein was 5.62 ± 0.69 mg.
(TIF)

S6 Fig SDS-PAGE analysis of lipase from metagenomic SeqB. Lane M: protein molecular weight marker; Lane F1 and F2: unbounded protein fractions; Lane E1: Eluted protein with 100 mM Imidazole; Lane E2: Eluted protein with 200 mM Imidazole and Lane E3: Eluted protein with 250 mM Imidazole. The partially purified enzyme was indicated by arrow. The total protein concentration of eluted protein was 0.699 ± 0.037 mg.
(TIF)

S1 Raw images (PDF)
