# Supplementary material for: Metagenomic discovery of lipases with predicted structural similarity to Candida antarctica lipase B
Source: PLoS One. 2023 Dec 6;18(12):e0295397. doi: 10.1371/journal.pone.0295397 (PMC10699602; doi:10.1371/journal.pone.0295397)
Supplement: S1 Table — (DOCX) [file pone.0295397.s001.docx]

**S1 Table.** **Example of metagenomics proteins having sequence identities with CalB lower than 30%.**

| **ID** | **Length** | **Best matching hit in BLAST search (NR database)** | | | **Protein alignment with CalB** | | | **RMSD between Alphafold predicted structure and CalB** | |
| --- | --- | --- | --- | --- | --- | --- | --- | --- | --- |
|  |  | **Description** | **Identities** | **Identities** | | **Similarity** |  | |  |
| MGS-1 | 356 | lipase [*Marmoricola sp*.] | 189/307 (62%) | 109/361 (30.2%) | | 150/361 (41.6%) | 3.44 | |  |
| MGS-2 | 356 | lipase [*Pseudonocardia spinosispora*] | 248/306 (81%) | 107/366 (29.2%) | | 157/366 (42.9%) | 3.42 | |  |
| MGS-3 | 338 | lipase [*Acidimicrobiales bacterium*] | 317/320 (99%) | 97/347 (28.0%) | | 150/347 (43.2%) | 3.44 | |  |
| MGS-4 | 244 | MAG: lipase [*Actinomycetota bacterium*] | 134/234 (57%) | 70/349 (20.1%) | | 104/349 (29.8%) | 4.5 | |  |
| MGS-5 | 133 | lipase [*Actinomycetota bacterium*] | 82/130 (63%) | 43/333 (12.9%) | | 66/333 (19.8%) | 4.19 | |  |
| MGS-6 | 193 | lipase [*Salinisphaeraceae bacterium*] | 119/178 (67%) | 54/358 (15.1%) | | 83/358 (23.2%) | 10 | |  |
| MGS-7 | 116 | Lipase B [*Teratosphaeria destructans*] | 58/86 (67%) | 37/330 (11.2%) | | 62/330 (18.8%) | 4.22 | |  |
| MGS-8 | 179 | lipase [*Blastococcus* sp. TF02-8] | 73/118 (62%) | 42/388 (10.8%) | | 70/388 (18.0%) | 20.8 | |  |
