# Supplementary figures and images for: Metagenomic discovery of lipases with predicted structural similarity to Candida antarctica lipase B
Source: PLoS One. 2023 Dec 6;18(12):e0295397. doi: 10.1371/journal.pone.0295397 (PMC10699602; doi:10.1371/journal.pone.0295397)

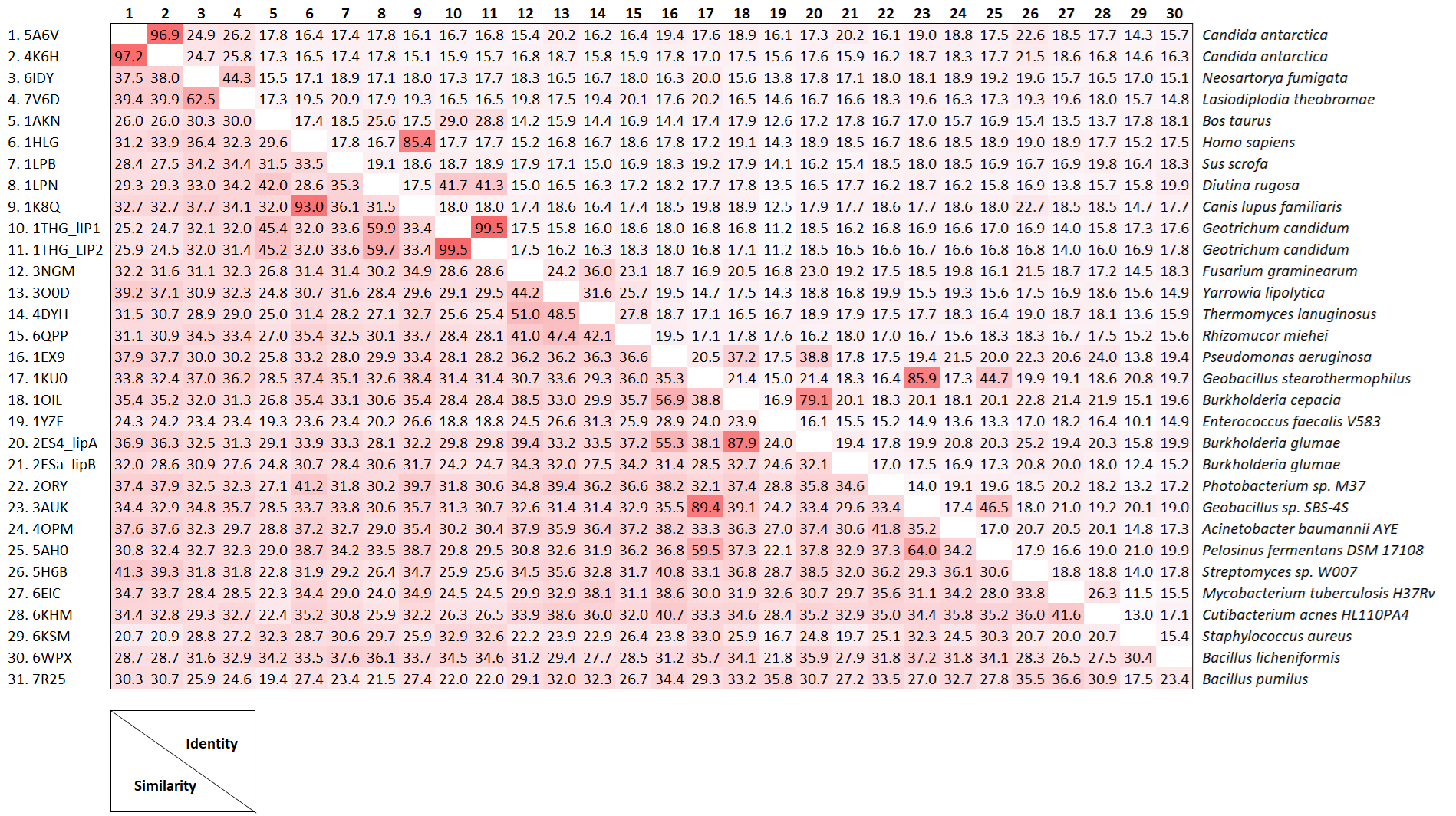

Supplement: S1 Fig — Data on the upper right presents the sequence identity and data on the bottom left presents the sequence similarity. The multiple sequence alignment was created by MatGAT2. The sequence no.1 to 15 are lipases from eukaryotes and no.16 to 31 are lipases from prokaryotes. (TIF) [file pone.0295397.s002.tif]

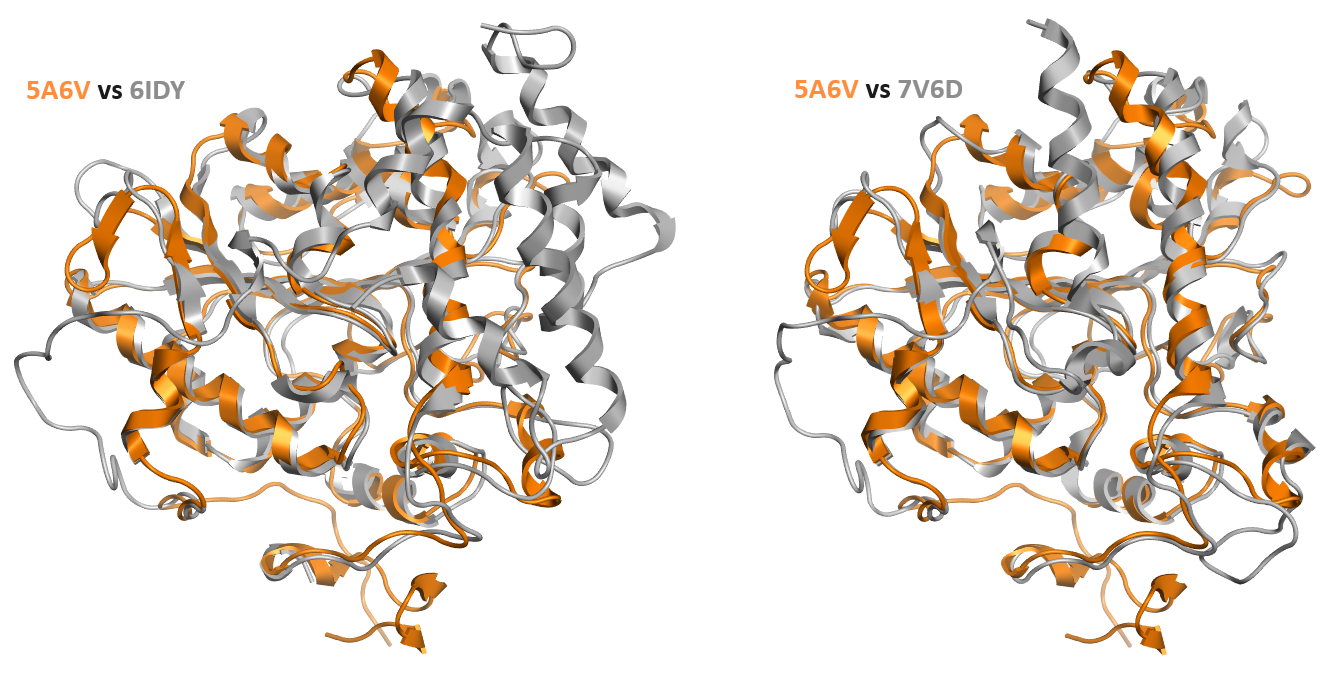

Supplement: S2 Fig — Lipases from Neosartorya fumigata (PDB: 6IDY) and Lasiodiplodia theobromae (PDB: 7V6D) are presented in grey. CalB structure is presented in orange. (TIF) [file pone.0295397.s003.tif]

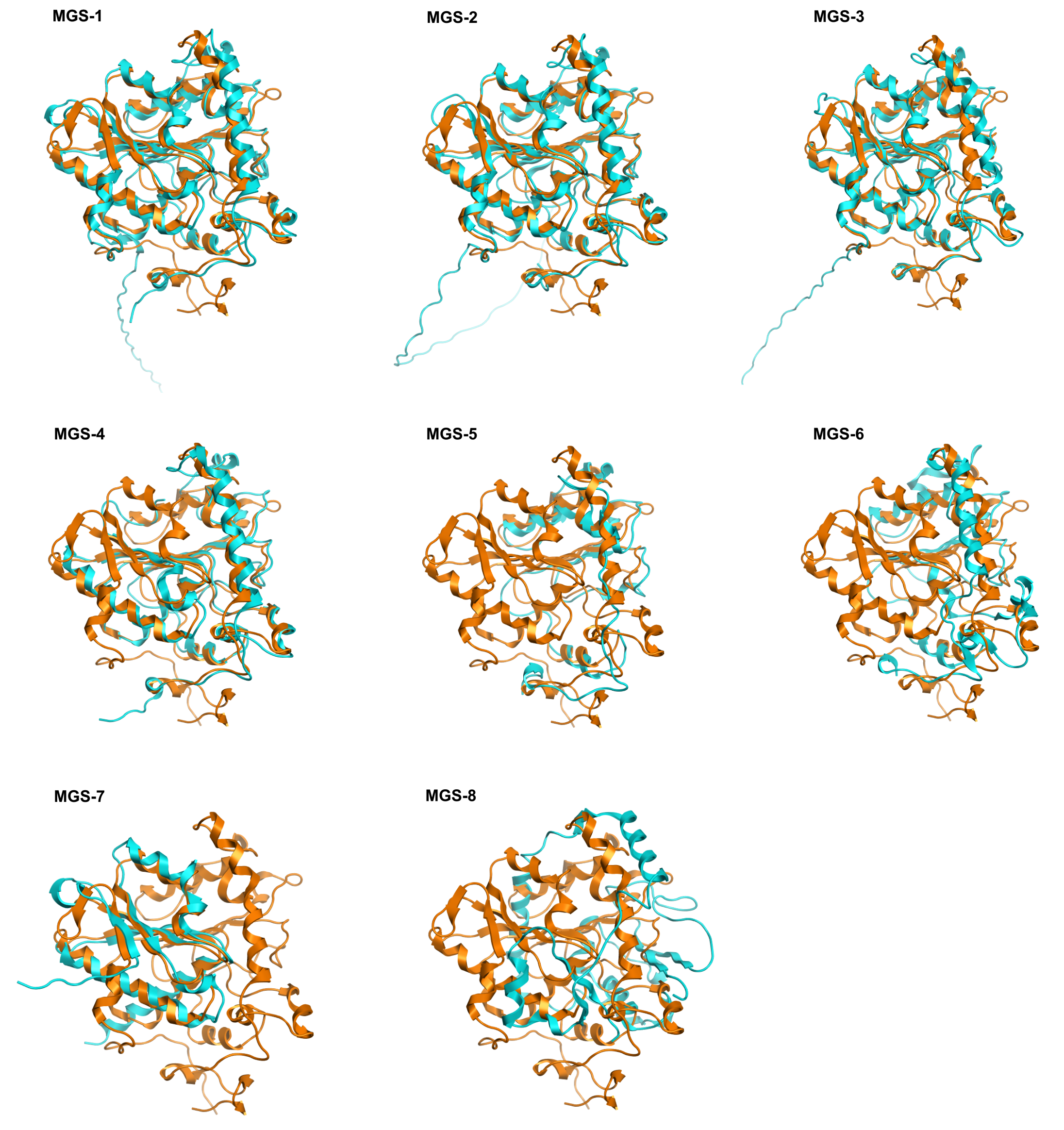

Supplement: S3 Fig — CalB structure is presented in orange and predicted structures of metagenomic lipases are presented in cyan. (TIF) [file pone.0295397.s004.tif]

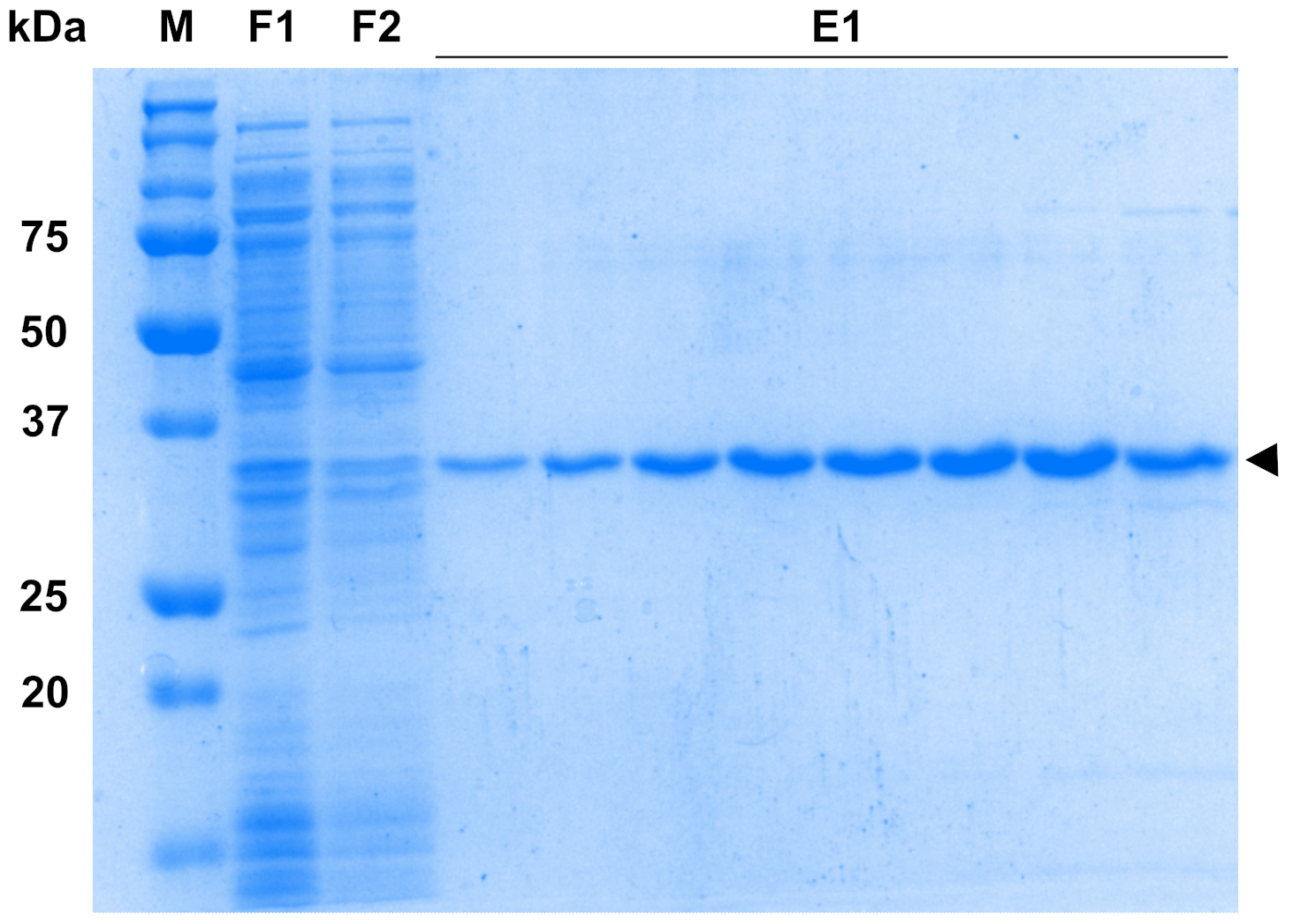

Supplement: S4 Fig — Lane M: protein molecular weight marker; Lane F1 and F2: unbounded protein fractions; Lane E1: Eluted protein with 100 mM Imidazole. The partially purified enzyme was indicated by arrow. The total protein concentration of eluted CalB protein was 5.06 ± 0.41 mg. (TIF) [file pone.0295397.s005.tif]

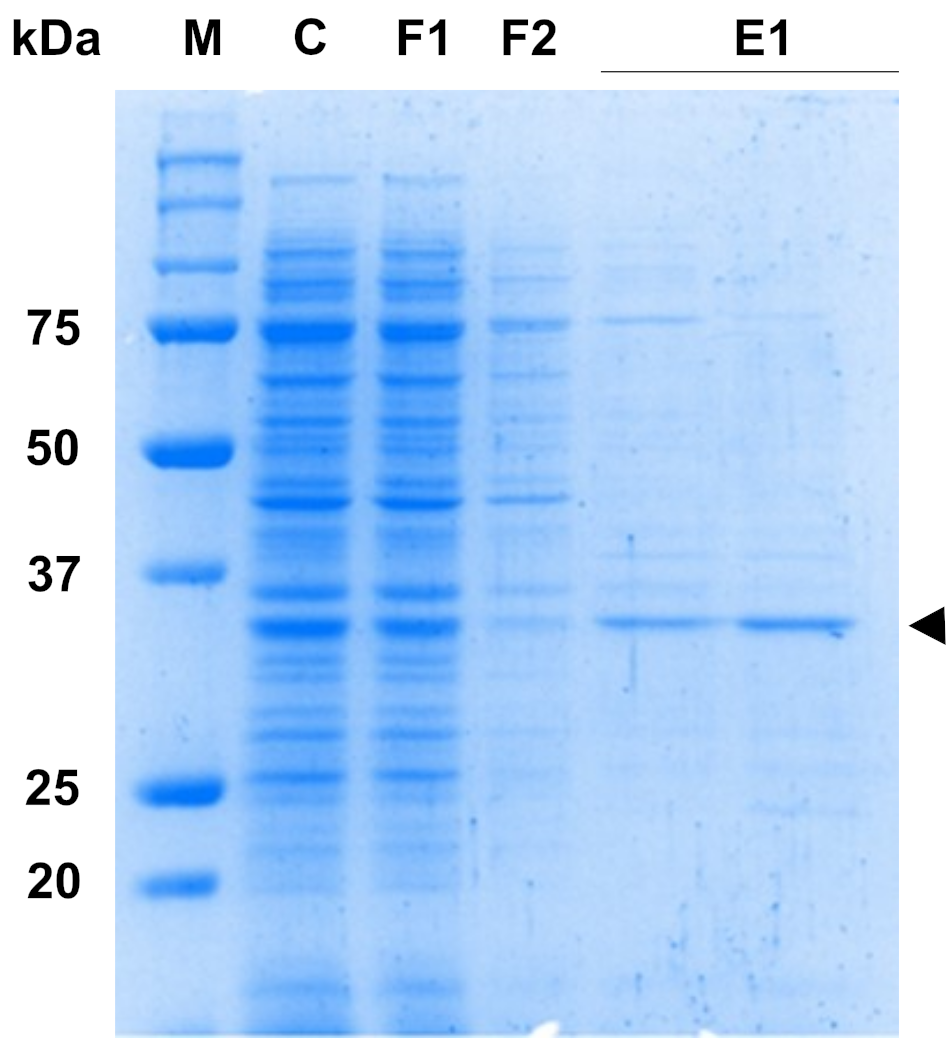

Supplement: S5 Fig — Lane M: protein molecular weight marker; Lane C: crude enzyme extract; Lane F1 and F2: unbounded protein fractions; Lane E1: Eluted protein with 100 mM Imidazole. The partially purified enzyme was indicated by arrow. The total protein concentration of eluted protein was 5.62 ± 0.69 mg. (TIF) [file pone.0295397.s006.tif]

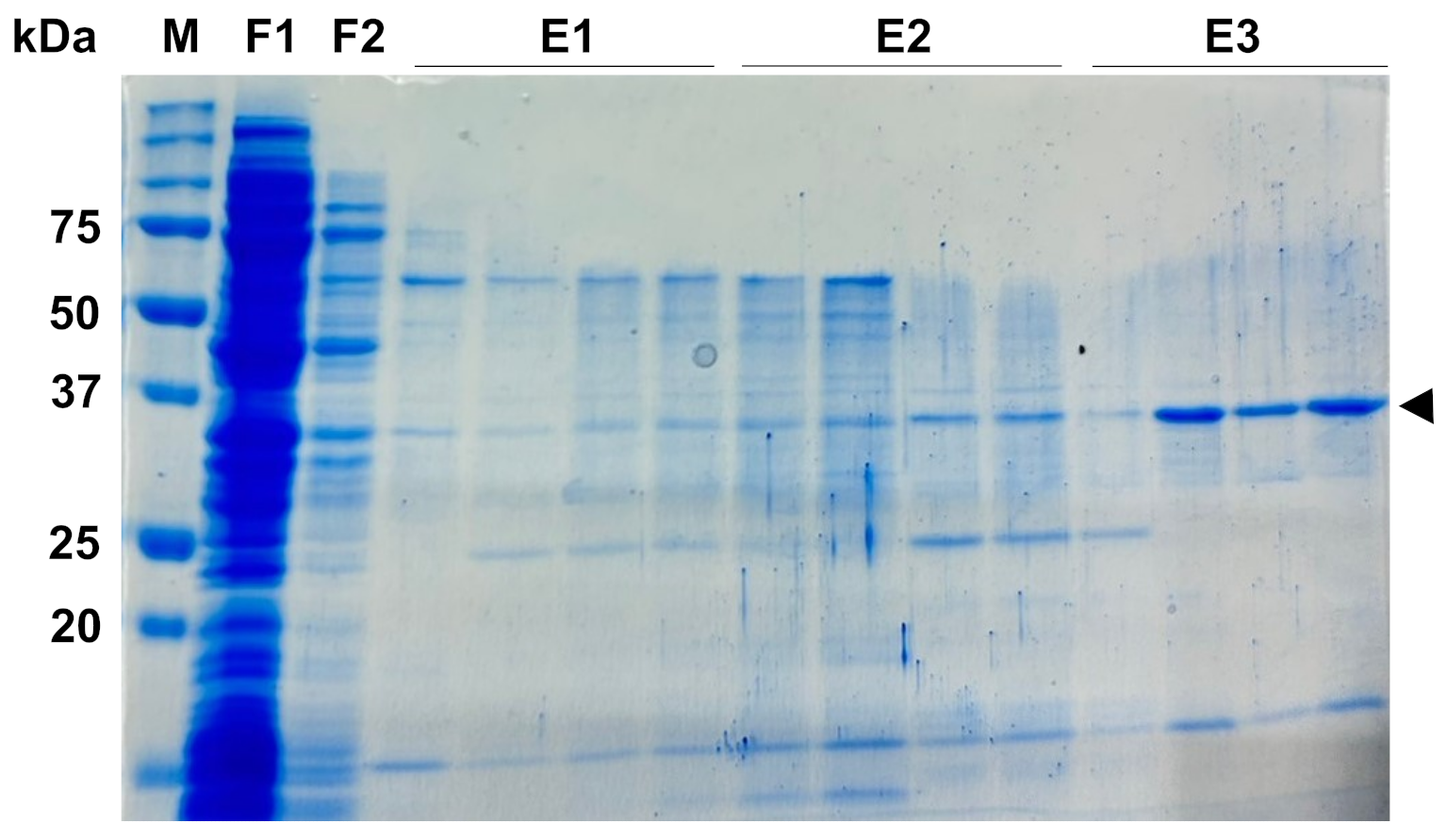

Supplement: S6 Fig — Lane M: protein molecular weight marker; Lane F1 and F2: unbounded protein fractions; Lane E1: Eluted protein with 100 mM Imidazole; Lane E2: Eluted protein with 200 mM Imidazole and Lane E3: Eluted protein with 250 mM Imidazole. The partially purified enzyme was indicated by arrow. The total protein concentration of eluted protein was 0.699 ± 0.037 mg. (TIF) [file pone.0295397.s007.tif]

S4\_raw\_image

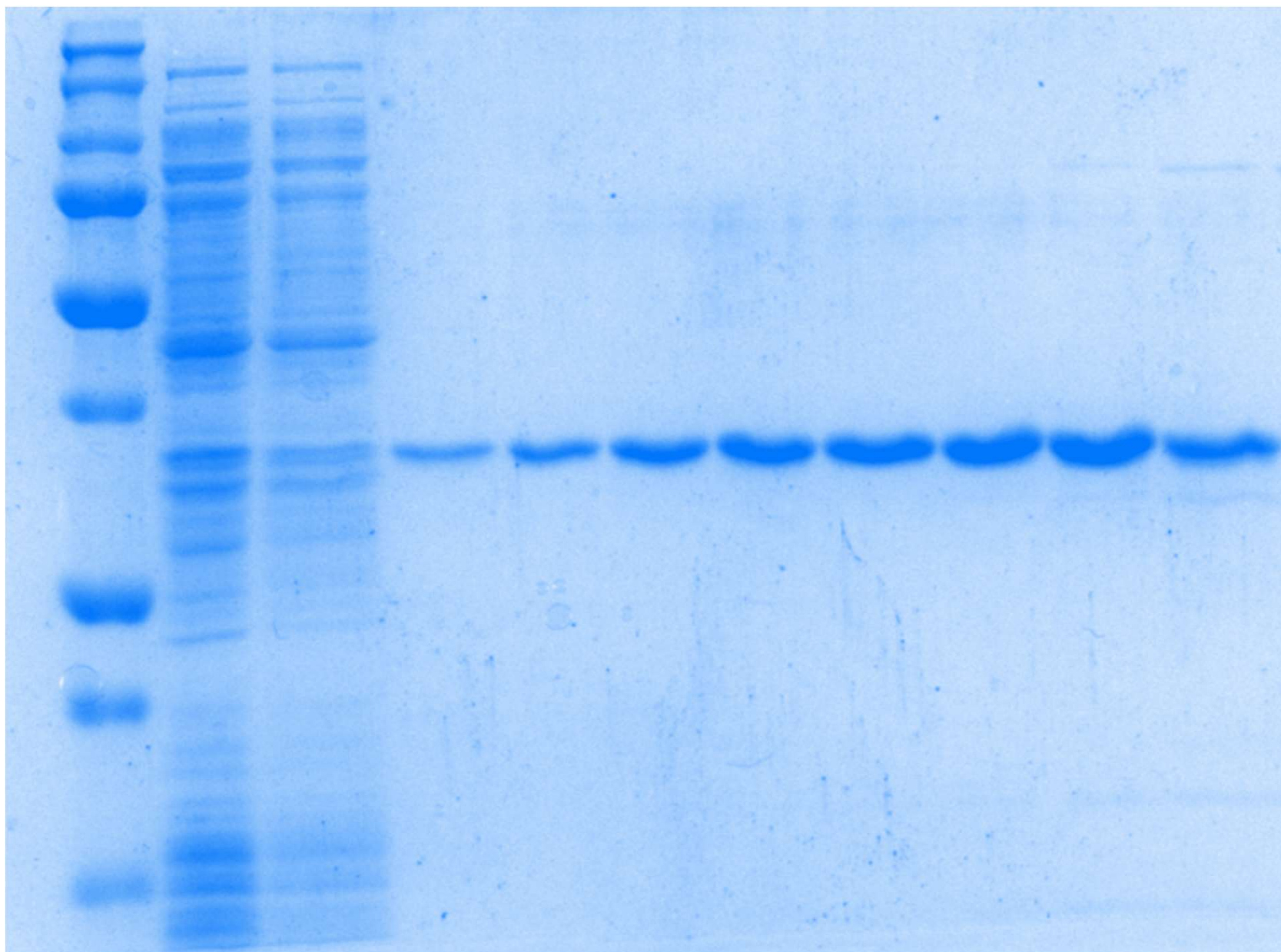

S5\_raw\_image

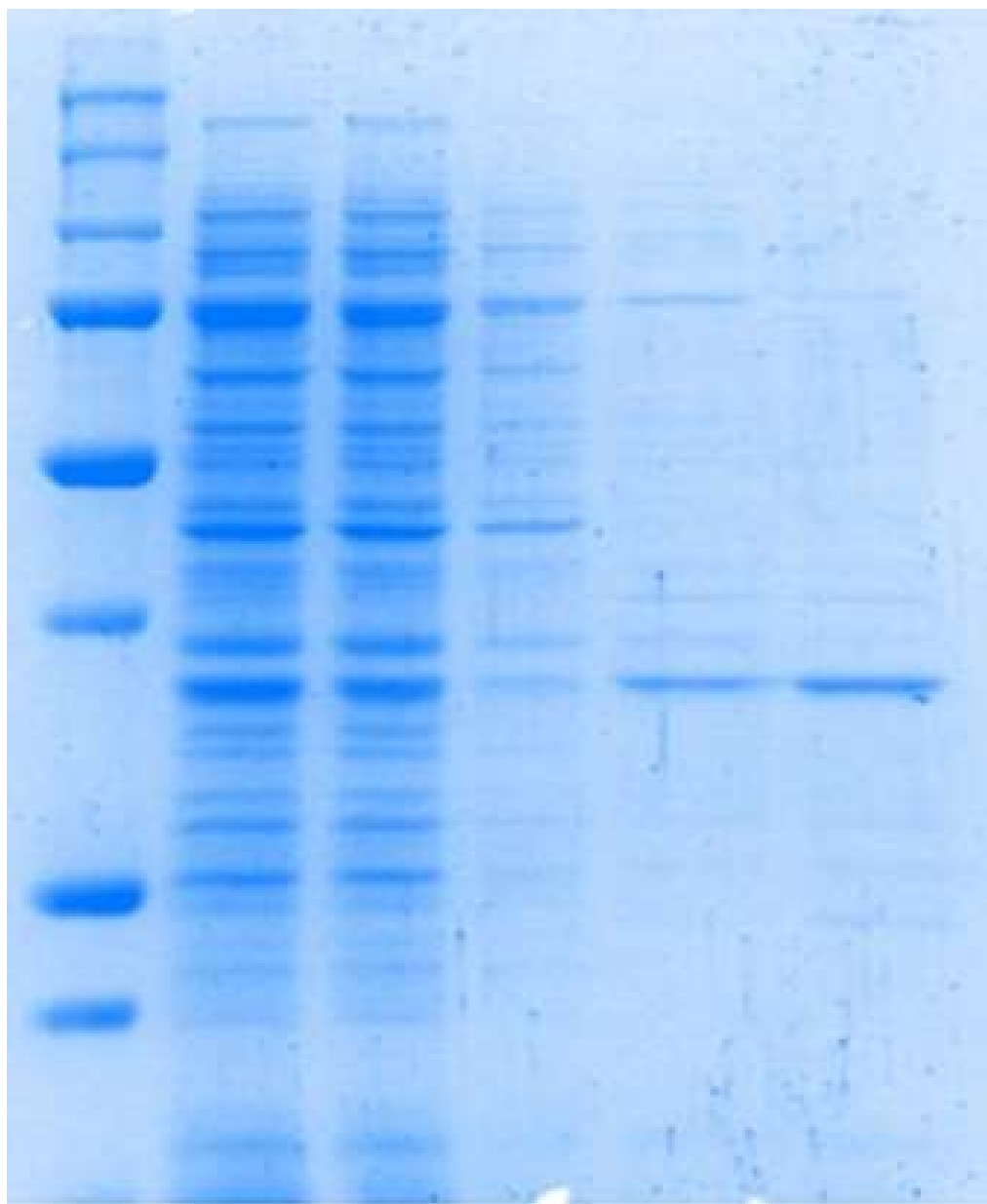

S6\_raw\_image

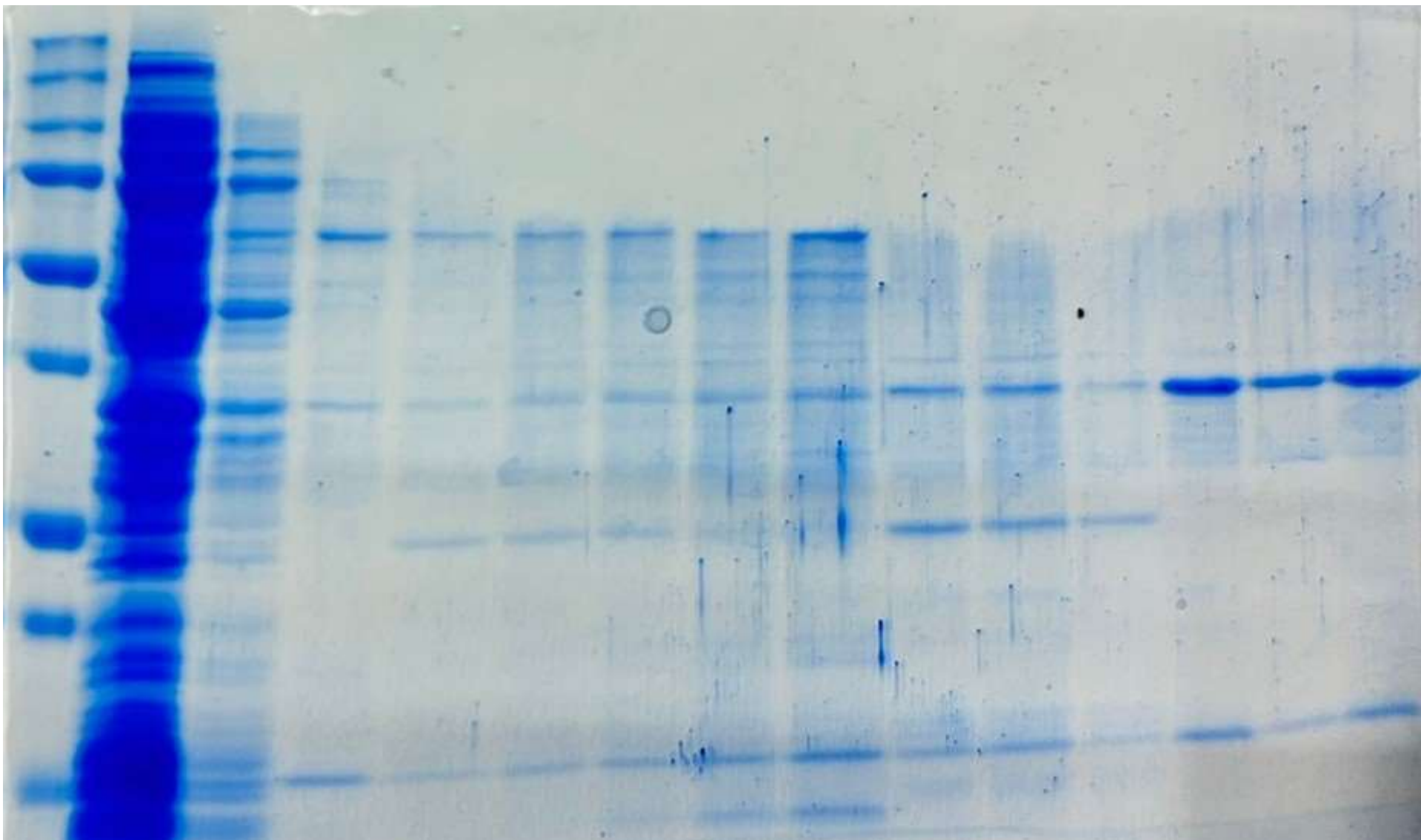

Supplement: S1 Raw images — (PDF) [file pone.0295397.s008.pdf]
